# Analyzing free fatty acids in seminal plasma from asthenozoospermia patients undergoing antioxidant therapy

**DOI:** 10.5935/1518-0557.20240086

**Published:** 2025

**Authors:** Naser Amirjannati, Mahdieh Aghabalazadeh Asl, Elham Hosseini, Ralf Henkel, Niloofar Agharezaee, Raheleh Kafaeinezhad, Hassan Rezadoost, Kambiz Gilany

**Affiliations:** 1 Department of Andrology and Embryology, Reproductive Biotechnology Research Center, Avicenna Research Institute, ACECR, Tehran, Iran; 2 Department of Phytochemistry, Medicinal Plants and Drugs Research Institute, Shahid Beheshti University, Tehran, Iran; 3 Zanjan Metabolic Diseases Research Center, Zanjan University of Medical Sciences, Zanjan, Iran; 4 Department of Obstetrics and Gynecology, Mousavi Hospital, School of Medicine, Zanjan University of Medical Sciences, Zanjan, Iran; 5 LogixX Pharma, Theale, Berkshire, United Kingdom; 6 Department of Medical Bioscience, University of the Western Cape, Bellville, South Africa; 7 Department of Metabolism, Digestion and Reproduction, Imperial College London, London, United Kingdom; 8 Monoclonal Antibody Research Center, Avicenna Research Institute (ACECR), Tehran, Iran; 9 Department of Bioinformatics, Kish International Campus University of Tehran, Kish, Iran; 10 Department of Biology, Faculty of Basic Sciences, University of Maragheh, Maragheh, Iran; 11 Integrative Oncology Department, Breast Cancer Research Center, Motamed Cancer Institute, ACECR, Tehran, Iran

**Keywords:** asthenozoospermia, free fatty acid, antioxidant therapy, seminal plasma

## Abstract

**Objective:**

Different aspects of the functions of free fatty acid (FFA) in seminal plasma and their implications on male fertility are known. However, the profile of FFA in seminal plasma in asthenozoospermic patients following antioxidant therapy has not been studied.

**Methods:**

In this case-control study, the total antioxidant capacity (TAC) and FFA profile of the seminal plasma were determined in 80 patients (29 normozoospermic volunteers and 51 asthenozoospermic men) who were treated with antioxidants for three months.

**Results:**

The TAC level in normozoospermic men was significantly higher than in asthenozoospermic men before and after antioxidant therapy with even lower values after the treatment (p=0.0001). The most abundant identified FFAs in seminal plasma were palmitic acid, vaccenic acid, eicosatrienoic acid, stearic acid, and myristoleic acid. Palmitic acid was lower in asthenozoospermic patients (*p*=0.0001), and antioxidant treatment restored its level to near-control levels. Compared to normozoospermic controls, the level of eicosatrienoic acid is significantly lower in asthenozoospermia patients before (*p*=0.01) and after treatment (*p*=0.0001). Additionally, following oral antioxidant supplementation, the FFA pattern in asthenozoospermic patients changes to the pattern observed in normozoospermic men. However, these changes are not statistically significant.

**Conclusions:**

The TAC level in asthenozoospermic patients after antioxidant treatment did not change to the levels in the control group; it even dropped to a lower level following three months of treatment. Antioxidant treatment can change the level of the FFA compositions of seminal plasma.

## INTRODUCTION

Men exhibiting normal sperm parameters but facing challenges in fathering a child constitute 15-30% of male infertility cases (Salas-Huetos *et al*., 2016). Seminal plasma (SP), a biological fluid originating from the male accessory sex glands, accompanies spermatozoa and apart from providing a protective environment for spermatozoa, SP encompasses crucial modulators influencing spermatozoa function (Wang *et al*., 2020). The significant functions of seminal plasma, traditionally confined to sperm transport and protection, have been historically undervalued. These developments offer new insights into various aspects of sperm activity, the fertilization process, and pregnancy outcomes. Traditionally, public knowledge regarding a men’s role in successful fertilization was confined to factors like sperm concentration, motility, and morphology. Consequently, evaluating male factor infertility relies on standard semen analysis according to WHO guidelines (WHO, 2021) with sperm parameters such as low number or absence of sperm, percentage of motility, and abnormal morphology. However, a standard semen analysis, despite enormous efforts by the WHO, suffers from poor standardization and subjectivity and is inadequate in accurately diagnosing male fertility potential (Wang & Swerdloff, 2014). Therefore, additional, more advanced and better standardizable parameters are necessary. Meanwhile, in the 6^th^ Edition of the WHO Laboratory Manual, sperm parameters such as DNA fragmentation or oxidative stress are recommended as extended examination and advanced examination, respectively (WHO, 2021). However, since seminal plasma is a very rich source of amino acids (Amirjannati *et al*., 2023) proteins (Rolland *et al*., 2013), polyamines (Vallely *et al*., 1988) and others including fatty acids (FAs), which could be possible biomarkers for male fertility, it has attracted significant interest due to the growing demand for the development of new tests to detect male factor infertility (Kumar & Singh, 2020). Seminal plasma, which is derived primarily from seminal vesicles (about 60%) and the prostate (about 30%), has been recognized as a valuable repository of a diverse set of heterogeneous metabolites, including sugars (fructose), lipids, proteins, ions, and cell-free nucleic acids. These molecular compositions of seminal plasma play important roles in the maturation, motility, function, and even nutrition of sperm cells (Wang *et al*., 2020; Chen *et al*., 2023; Grosso *et al*., 2021). Lipid components, in particular FAs, have drawn interest among the seminal plasma compositions and plasma membrane of spermatozoa for their potential effects on sperm nutrition and fertility function (Zerbinati *et al*., 2016).

FAs, whether existing as single molecules or as part of larger molecular structures, serve diverse biological roles. These roles span from contributing to the composition of cell membranes to acting as providers of energy and signaling molecules (Deckelbaum & Torrejon, 2012; Collodel *et al*., 2020a; de Carvalho & Caramujo, 2018). Interestingly, spermatozoa have a variety of enzyme resources for oxidizing fatty acids and ATP generation, as evidenced by the suppression of oxidation by etomoxir resulted in a reduction in sperm motility. Along with glycolysis, the Krebs cycle, sperm need fatty acids of various chain lengths, supplying power to the movement of sperm (Amaral *et al*., 2013; Peña *et al*., 2022). Due to the extreme polarization of spermatozoa and their special functions, sperm plasma membranes contain an extraordinarily high amount of polyunsaturated fatty acids (PUFA) (Parks & Lynch, 1992), a fact which makes spermatozoa particularly susceptible to oxidative assaults through leukocytes or oxidative stress (Henkel, 2011).

Zerbinati *et al*. (2016) reported a significant difference in FA distribution in the seminal plasma of asthenozoospermic and oligoasthenoteratozoospermic patients compared to men with normozoospermia. In addition, these authors identified four FAs, namely palmitic acid, behenic acid, oleic acid, and docosahexaenoic acid, as promising indicators with diagnostic and therapeutic potential for sperm quality to be used as targets of tailored dietary/supplement therapies in individuals with particular changes in FA profile (Zerbinati *et al*., 2016).

Martínez-Soto *et al*. (2013) investigated possible links between the fatty acid content of human spermatozoa and seminal plasma before freezing and sperm parameters (motility and viability) before and after the freezing-thawing process. The study showed that sperm parameters following thawing were directly correlated with PUFAs, ω3 PUFAs, and docosahexaenoic acid (DHA) of spermatozoa. In contrast, an inverse relationship was found for monounsaturated fatty acids (MUFA), and the ratio of saturated fatty acids (SFA)/PUFA. On the other hand, the FA content of seminal plasma was related to sperm motility, but not viability (Martínez-Soto *et al*., 2013).

Asthenozoospermia refers to a condition characterized by reduced sperm motility (<42% total motility, <30% progressive motility) according to the reference range of the 6^th^ Edition of the WHO laboratory manual (WHO, 2021), and is one of the factors contributing to male infertility. In asthenozoospermic patients, antioxidant therapy is employed as one of the treatment approaches, given the implication of oxidative stress in sperm dysfunction (Balercia *et al*., 2009; Kaltsas, 2023). Individual reactions to antioxidant therapy can vary, and the overall effectiveness of such intervention can be influenced by several factors, including the specific antioxidants applied, the duration of treatment, and the underlying reasons for asthenozoospermia (Kaltsas, 2023). Vitamins are integral coenzymes in lipid metabolism, facilitating fatty acid synthesis and oxidation as hypovitaminosis may contribute to lipid metabolism disorders, while certain vitamins exhibit pharmacological effects by modulating lipoprotein fractions (McNeil *et al*., 2012; Fidanza & Audisio, 1982; Saraswathy *et al*., 2018).

Recent investigations have explored various aspects of FA function in male reproduction, including their metabolism in spermatogenesis, the impact of dietary FAs on the sperm FA profile, the significance of FA composition in sperm quality, the relationship between FA composition and sperm parameters, and the possible implications of FA composition on male reproductive status (Collodel *et al*., 2020a; 2020b; Nassan *et al*., 2018). Several studies have already investigated the seminal plasma metabolomics profile in different groups of patients (Correnti *et al*., 2023; Boguenet *et al*., 2020). However, to the best of our knowledge, no research has been conducted on the FA profile of seminal plasma in patients with sperm parameter complications following antioxidant therapy. Therefore, this study aimed to investigate the FA composition in seminal plasma of asthenozoospermic patients receiving antioxidant therapy and compare these to normozoospermic men.

## MATERIAL AND METHODS

### Study population

This was a case-control, before/after study of 80 patients, including 29 normozoospermic volunteers and 51 asthenozoospermic patients, between January 2020 and December 2020 in the Urology Department of the Avicenna Fertility Center (associated with Avicenna Research Institute, Tehran, Iran).

The normozoospermic men were considered non-alcoholic and non-smoking volunteers who had fathered at least one child previously and were referred for sex selection family balancing. All asthenozoospermic men included in this study had a minimum of 1 year of regular unprotected intercourse. Patients who had taken antioxidant supplements in the last three months were excluded. Furthermore, patients who had to receive other medications during antioxidant therapy were also excluded from the study.

The study was approved by the Ethics Committee of the Avicenna Research Institute (ARI) (IR.ACECR.Avicenna.REC.1395.4).

### Sample collection

Age and BMI were recorded for all patients in the study, including normozoospermic volunteers and asthenozoospermic patients, with an age range of 25-50 and BMI less than 30. Semen samples were collected by masturbation in a sterile plastic container after 3-6 days of sexual abstinence. All the samples were allowed to liquefy at 37°C for 30 minutes and were subsequently assessed via light microscopy according to the World Health Organization (WHO) laboratory manual for the examination and processing of human semen (6^th^ Ed., 2021). The following variables were taken into consideration: ejaculate volume (mL), pH, sperm concentration (10^6^ sperm per mL), total sperm count (10^6^ sperm per ejaculate), progressive motility (%), and normal morphology (%). The subjects were classified according to their sperm parameters into Group 1 (normozoospermic, n=29), Group 2 (asthenozoospermic before antioxidant supplementation, n=51), and Group 3 (asthenozoospermic participants after antioxidant supplementation, ASTAntiOxSupp, n=51).

Following semen analysis, 1 mL of the remaining semen samples were frozen at -80°C until further analysis.

### Antioxidant Supplementations

In this study, asthenozoospermic patients were treated with vitamin E 400 IU/day + selenium 60 mg/day + folic acid 5 mg/day for a period of 3 months. Spontaneous pregnancies in the study group (asthenozoospermic patients) were assessed after 3 months of supplementation and during a 6-months follow-up period.

### Total Antioxidant Capacity

The seminal plasma’s total antioxidant capacity (TAC) was assessed following established procedures (Amirjannati *et al*., 2023). In brief, an antioxidant assay kit (Dianbioassay, Tehran, Iran) was employed to determine TAC based on the seminal plasma antioxidants’ ability to hinder the oxidation of ABTS (2,20-azino-di-[3-ethylbenzthiazoline sulfonate]) to ABTS+. Briefly, 50 µl of SP was thawed and diluted 1:10 with deionized water. Then, 20 µl of thawed SP was added to 180 µl of reagent I, and the absorbance was measured at 660 nm. Thereafter, 20 µl of reagent II was added and incubated in the dark for 10 minutes, after which the absorbance was read at 660 nm. The difference between the first and second absorbance measurements was calculated. Finally, the TAC value of the SPs was determined in µmol using a standard curve.

### Analysis of Free Fatty Acids (FFA) in Seminal Plasma

For FFA analysis, 50 µL of seminal plasma was thawed for gas chromatography analysis. Briefly, FFAs were extracted based on the Folch method via the use of chloroform/methanol at a volumetric ratio of 2:1 (v/v). One hundred microliters of extraction buffer were added to 50 µL of SP. The sample was mixed and centrifuged for 15 min at 4000 rpm. The supernatant, which contains FFAs, was allowed to dry. The dried FFAs were first directly trans-folded by potassium hydroxide (KOH) followed by boron trifluoride (BF3) in methanol, which allows for both the derivatization of free and esterified FAs as methyl esters (FAME). The identities of the peaks were assigned using mixtures of authentic FAME standards (GLC-462; Nuchek Prep, Elysian, MN, USA). For FFA analysis, 1 µL of seminal plasma was injected into gas chromatography (GC). Analyses were performed on an Agilent 7890A Plus Gas Chromatograph (Agilent Technologies, Santa Clara, USA) equipped with a G4513A automatic liquid sampler and a flame-ionization detector (GC-FID). Separation was carried out on a 100-m capillary column (Agilent, CP-Sil 88 GC Columns, 100 m, 0.25 mm inner diameter, 0.20 µm thickness). The area percentages of each FAME were obtained. Finally, the data are shown as the percentage of FFAs by weight for samples of whole seminal fluid.

### Couple follow-up

Asthenozoospermic patients and their respective partners underwent an initial assessment on the commencement of antioxidant intervention, followed by subsequent evaluations at three-month intervals and, subsequently, at six-month intervals, to systematically monitor reproductive outcomes.

The serum human chorionic gonadotropin (hCG) levels were measured in the absence of menstruation to test for pregnancy. On transvaginal ultrasound scanning, a clinical pregnancy was defined as the existence of a gestational sac and fetal heartbeat.

### Statistical analysis

A descriptive analysis was conducted to evaluate the characteristics of the 80 participants. The data are presented as the mean and standard error of the mean (±SEM). The data were log10-transformed and normalized. The Kolmogorov‒Smirnov normality test was used to determine the normal distribution of a data set the data. For comparisons of age and body mass index (BMI) between the different groups of patients the Student’s t-test was used. ANOVA followed by post hoc Tukey’s test was used to determine significant differences between the groups. All the statistical analyses were performed with GraphPad Prism software version 8.4.3 (1992-2020 GraphPad Software, LLC, Boston, USA). A *p*-value of *p*<0.05 was considered significant.

## RESULTS

### Semen parameters

Twenty-nine normozoospermic volunteers as controls and 51 asthenozoospermic patients were enrolled in this study for free fatty acid analysis using gas chromatography and a flame-ionization detector GC-FID. Semen samples of the asthenozoospermic patients were analyzed before and after oral antioxidant supplementation (vitamin E 400 IU/ day + selenium 60 mg/day + folic acid 5 mg/day) for three months (ASTAntiOxSupp).

The demographic data, clinical features, and spermiogram parameters of all 80 participants are summarized in Table 1. The control (normozoospermic) and asthenozoospermic groups showed significant differences in age and BMI. The three groups did not differ in terms of abstention time, ejaculate volume, and pH. Significant differences were observed in terms of sperm concentration, total sperm count, and motility between the normozoospermic, asthenozoospermic, and ASTAntiOxSupp groups (*p*<0.0001). However, there were no significant changes in the semen parameters between groups 2 (asthenozoospermic before treatment) and group 3 (asthenozoospermic after treatment; ASTAntiOxSupp). Furthermore, normal sperm morphology in the three groups was in the normal range and the differences were not significant.

**Table 1:** Demographic and spermiogram and pregnancy data of partners of the volunteers.

Variable	Group 1 (n=29)	Group 2 (n=51)	Group 3 (n=51)	1vs.2vs.3	1 vs. 2	1 vs. 3	2 vs. 3
Age (years)	39.4±5.6	36.1±6.1			<0.05a		
BMI (kg/m^2^)	28.5±3.8	26.7±3.2			0.03		
Abstention time (day)	4.0±2	3.4±1	3.6±2				
**Semen Parameters** Volume (ml) pH Sperm concentration (106/ml) Total sperm count (106 /ejaculate) Progressive (%) Morphology (%)	3.8±1.9 7.5±0.1 45±2.9 170±5.5 41±2 5.8±0.4	3.2±1.2 7.5±0.1 32±2.8 96±3.4 25±2 4.1±0.4	3.3±1.3 7.5±0.1 35±2.4 105±3.1 28±2 4.3±0.3	0.0001b 0.0001 0.0001	0.0001 0.0004 0.0001	0.002 0.003 0.0001	n.s. n.s. n.s.
**Pregnancy data** Total pregnancy Clinical pregnancy rate Miscarriage rate	-	0/51 0/51 0/51	9/51 9/51 3/9				

The values are expressed as the means (±SEMs). Group 1, normozoospermic; Group 2, asthenozoospermic before antioxidant therapy; Group 3, asthenozoospermic after antioxidant therapy. (^a^) Student's t test was performed for two groups. (^b^) ANOVA and post hoc Tukey’s tests were performed for statistical analyses. BMI: body mass index.

### Total antioxidant capacity

**Figure 1:**
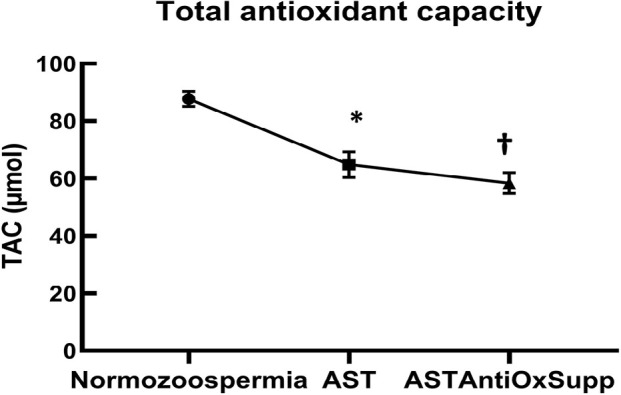
Total antioxidant capacity of seminal plasma following antioxidant supplementation. The values are expressed as the normalized means±SEMs. AST: asthenozoospermic, ASTAntiOxSupp: asthenozoospermic antioxidant supplement.

Figure 1 shows the TAC of the seminal plasma from normozoospermic, and asthenozoospermic patients before and after antioxidant therapy. The concentration of TAC was significantly higher in the normozoospermic controls compared to asthenozoospermic patients before and after antioxidant therapy (*p*=0.0001). However, although the mean value of normalized TAC of the asthenozoospermic patients before the antioxidant treatment was slightly higher than after the treatment, there was no significant difference (*p*=0.4).

### Fatty acid analysis

**Figure 2:**
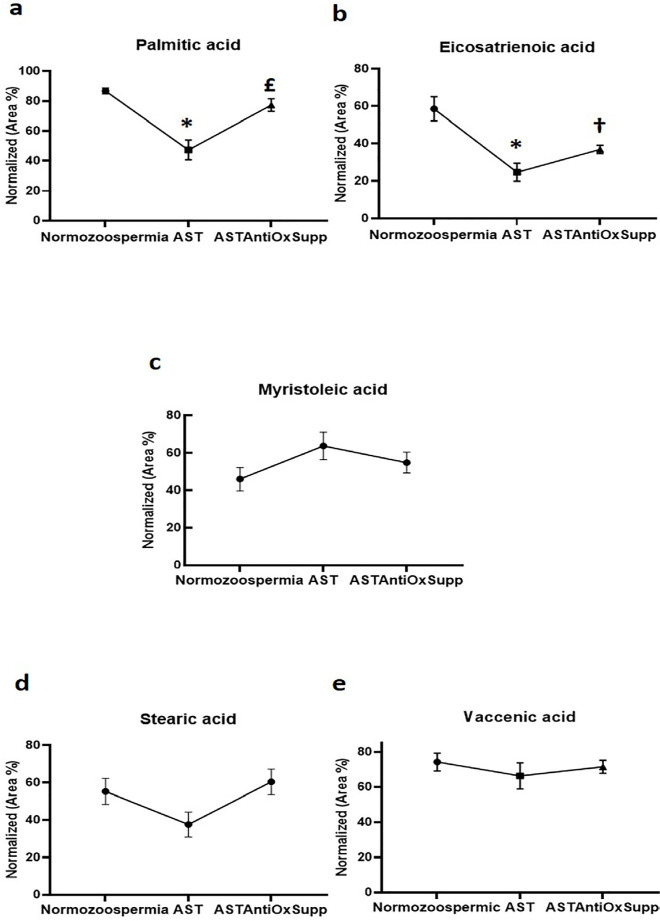
The pattern of palmitic acid **(a)**, eicosatrienoic acid **(b)**, myristoleic acid **(c)**, stearic acid **(d)**, and vaccenic acid **(e)** levels in normozoospermic patients (n=29) and following antioxidant therapy in asthenozoospermic patients (n=51). The values are expressed as the normalized mean (±SEM) area percentage. AST, asthenozoospermic; ASTAntiOxSupp, asthenozoospermic after antioxidant supplementation.

Preliminary injection of 28 fatty acids by GC-FID allowed the identification and quantification of 5 FFAs in the panel using 1 µL of seminal plasma. The most abundant identified FFAs in the seminal plasma were palmitic acid, vaccenic acid, eicosatrienoic acid, stearic acid, and myristoleic acid (Figure 2).

* Significant difference between normozoospermia and AST

£ Significant difference between AST and ASTAntiOxSupp

† Significant difference between normozoospermia and ASTAntiOxSupp.

Figure 2a shows the levels of palmitic acid in the studied groups. Palmitic acid had a significantly (*p*=0.0001) lower concentration in the SP of asthenozoospermic patients than in the controls. After antioxidant treatment, its levels were significantly (*p*= 0.0001) restored to near-control levels.

Figure 2b shows that the level of eicosatrienoic acid is significantly lower in asthenozoospermic patients before (*p*=0.0001) and after (*p*=0.01) antioxidant therapy compared to normozoospermic subjects. Additionally, after antioxidant therapy, the level of eicosatrienoic acid increased. However, this increase is not significant (*p*=0.2).

Figure 2c shows the level of myristoleic acid in the studied groups. The myristoleic acid level is higher in asthenozoospermic men than in normozoospermic samples. Following oral antioxidant supplementation, the levels of myristoleic acid decreased to levels between those of normozoospermic and asthenozoospermic men. Yet, these changes are not statistically significant.

Figures 2d and 2e show the levels of stearic acid and vaccenic acid in the normozoospermic and asthenozoospermic before and after antioxidant supplementation. Levels of both fatty acids are lower in asthenozoospermic men compared to normozoospermic subjects. After antioxidant treatment, the levels of these fatty acids returned to values comparable to near-control levels. However, these changes are not statistically significant.

### Correlations of semen parameters with FFA levels

In this study, we conducted a correlation analysis between semen parameters, including concentration, count, motility, and morphology, and identified FFA levels. There were no statistically significant correlations between sperm parameters and identified FFAs.

### Clinical outcome

As indicated in Table 1, over the six-month follow-up period post-antioxidant treatment, 9 out of 51 female partners achieved pregnancy (pregnancy rate = 17.6%), in contrast to the pre-treatment phase (0/51).

## DISCUSSION

To the best of our knowledge, our study is the first to provide evidence of the consequences of oral antioxidant supplementation on the fatty acid composition of seminal plasma of asthenozoospermia patients.

There is growing evidence suggesting that the fatty acid composition of seminal plasma could be a determining factor for male fertility (Collodel *et al*., 2022). On the other hand, the effect of oral antioxidant supplementation on semen parameters, particularly sperm motility has repeatedly been shown (Barbonetti *et al*., 2024; Busetto *et al*., 2018; De Leo *et al*., 2022). However, no study has established the optimal dose or duration of treatment for asthenozoospermic patients who might benefit most from oral antioxidant therapy (Zini & Al-Hathal, 2011). We did not observe a statistically significant improvement in semen parameters following oral antioxidant supplementation. This could be caused by the lack of an optimal dose or duration of the medication. This is further reflected in our measurement of TAC. The TAC level in the group of treated asthenozoospermic patients did not improve to the levels determined in the normozoospermic group; it even dropped to a lower level following three months of treatment.

There are several scenarios where a decrease in TAC might occur after antioxidant therapy. Firstly, the interplay of different antioxidants in the body is complex. In some cases, the introduction of exogenous antioxidants may disrupt the intricate balance of endogenous antioxidants, potentially leading to unexpected outcomes (Poljsak & Milisav, 2012). The duration and dosage of antioxidant therapy (specifically vitamin E) could also influence its effects on TAC. In some cases, excessive antioxidant supplementation might overwhelm the body’s natural regulatory mechanisms, resulting in unanticipated responses (Poljsak & Milisav, 2012; Miller *et al*., 2005). Finally, individuals may respond differently to antioxidant therapy which may be caused by variations in diet and lifestyle. Factors such as baseline TAC levels, overall health, and genetic variations could contribute to variations in the response to treatment (Vassalle *et al*., 2020).

Henkel *et al.* (2019) argued that overuse of antioxidant supplements can cause reductive stress, thus resulting in infertilit. Most recently, the European Society of Human Reproduction and Embryology (ESHRE) published evidence-based recommendations focusing on the safety and efficacy of antioxidant therapy. Since there is a lack of knowledge and evidence about the ideal antioxidant therapy, substantial and reliable evidence demonstrating a significant improvement in live birth rate, and their efficacy, the ESHRE has not recommended antioxidant therapy in male infertility treatment (ESHRE, 2023).

In the present study, we identified two SFAs (palmitic acid and stearic acid), two MUFAs (myristoleic acid and vaccenic acid), and one PUFA (eicosatrienoic acid) in semen samples. The level of palmitic acid changes to the level seen in normozoospermic men after three months of oral antioxidant supplementation. Palmitic acid is one of the most abundant SFAs in mammalian spermatozoa and plays an important role in fertilization (Esmaeili *et al*., 2015). It is suggested that vitamin E prevents oxidation of palmitic acid, thereby preventing damage induced by lipid peroxidation (Valk & Hornstra, 2000; Babenko *et al*., 2012; Khosrowbeygi & Zarghami, 2007). Furthermore, when added to the extender, palmitic acid was found to enhance bull sperm progressive linear motility via sperm mitochondrial activity and viability which suggests that mitochondrial β-oxidation, for which exogenous fatty acids serve as the primary energy source, is crucial for sperm progressive motility and survivability (Islam *et al*., 2021). Boar sperm also utilize palmitic acid as substrates for ATP generation through the mitochondrial β-oxidation pathway, serving as sources of energy (Zhu *et al*., 2020). The observed lower level of palmitic acid in asthenozoospermic patients in the present study underscores the potential role of lipid metabolism in sperm motility. The restoration of palmitic acid levels to near-control levels with antioxidant treatment may suggest a potential link between oxidative stress and lipid dysregulation in sperm.

Many studies have investigated the pivotal role of PUFAs, including arachidonic acid, linoleic acid, eicosapentaenoic acid (EPA), and docosahexaenoic acid (DHA), and their metabolites in sperm biology and spermatogenesis (Chen *et al*., 2023). The level of the identified PUFA eicosatrienoic acid was significantly lower in the asthenozoospermic group than in the normozoospermic group. This finding aligns with the results published by Safarinejad *et al.* (2010), who showed a low level of eicosatrienoic acid in infertile men. It is suggested that eicosatrienoic acid is the main ω3-source in the sperm plasma membrane and is essential for fertilization (Masoudi & Dadashpour Davachi, 2021). Following oral antioxidant therapy, a higher level of eicosatrienoic acid in asthenozoospermic patients was observed. Vitamin E has been reported to be a key essential lipophilic antioxidant in humans that protects PUFAs (Raederstorff *et al*., 2015).

MUFAs are suggested to defend against oxidative stress (Khalil *et al*., 2021). Myristoleic acid levels were increased in asthenozoospermia patients. However, after antioxidant supplementation, the concentration of this FA decreased again after three months of oral antioxidant supplementation. This could be caused by reductive stress, as suggested by Henkel *et al*. (2019). Furthermore, we identified another MUFA, vaccenic acid. It is shown that vaccenic acid decreases oxidative stress (Collodel *et al*., 2022; Collodel *et al*., 2021). As shown in our results, vaccenic acid showed lower levels (though not significant) in asthenozoospermic patients than in the normozoospermic controls. After antioxidant supplementation, the concentration increased again, almost reaching control levels.

Stearic acid is another SFA that was discovered in the present study. In the context of sperm biology, stearic acid has been suggested to be positively correlated with sperm motility (Zerbinati *et al*., 2016). Nevertheless, we could not establish any association with other semen parameters. On the other hand, stearic acid can protect against oxidative stress. It can be hypothesized that it plays an important role in the protection of sperm against oxidative stress via the phosphatidylinositol 3-kinase pathway (Wang *et al*., 2006). This can be seen in the upregulation of stearic acid in the seminal plasma after three months of oral antioxidant supplementation.

Nonetheless, most of our identified and quantified FAs were not significantly different. This could be due to small sample sizes or the difficulties in standardizing the diet and lifestyle as well as the variability of spermatozoa and seminal plasma (Collodel *et al*., 2022).

Furthermore, an attempt was made to correlate the identified and quantified FAs with the semen parameters. However, no statistically significant correlation was observed.

Moreover, we observed an improved pregnancy rate (9/51) after three months of oral antioxidant supplementation compared to before treatment (0/51). This finding follows the most recently published study by de Ligny *et al*. (2022), which showed an improved live birth rate after oral antioxidant supplementation independent of semen parameter quality in subfertile men.

## CONCLUSION

Several free fatty acids were identified and quantified from seminal plasma via GC-FID. Additionally, the pattern of fatty acid levels in seminal plasma from asthenozoospermic patients after antioxidant therapy improved toward that of the normozoospermic group. Antioxidant treatment appears to play a role in reinstating the balance of lipid profiles. These findings may have important clinical implications, suggesting a potential avenue for therapeutic interventions aimed at modulating lipid composition in seminal plasma.

## Ethics statement

The study involving human participants was reviewed and approved by the Ethics Committee of the Avicenna Research Institute (ARI) (IR.ACECR.Avicenna.REC.1395.4).
